# Diversification of Specificity after Maturation of the Antibody Response to the HIV gp41 Epitope ELDKWA

**DOI:** 10.1371/journal.pone.0031555

**Published:** 2012-02-14

**Authors:** Henry N. White, Qing-Hai Meng

**Affiliations:** Department of Molecular Immunology, University College London, Institute of Child Health, London, United Kingdom; INRA, France

## Abstract

During maturing antibody responses the increase in affinity for target antigens is achieved by genetic diversification of antibody genes followed by selection for improved binding. The effect this process has on the specificity of antibody for variants of the antigen is not well-defined, despite the potential role of antibody diversification in generating enhanced protection against pathogen escape mutants, or novel specificities after vaccination. To investigate this, a library of single amino-acid substitution epitope variants has been screened with serum obtained at different time-points after immunization of mice with the HIV gp41 peptide epitope ELDKWA. The serum IgG response is shown to mature and increase affinity for ELDKWA, and the titre and affinity of IgG against most epitope variants tested increases. Furthermore there is a bias towards high affinity serum IgG binding to variant epitopes with conservative substitutions, although underlying this trend there is also significant binding to many epitopes with non-conservative substitutions. Thus, maturation of the antibody response to a single epitope results in a broadening of the high-affinity response toward variant epitopes. This implies that many pathogen epitope escape variants that could manifest as single amino-acid substitutions would not emerge by escaping immune surveillance.

## Introduction

The relationship between serum antibody affinity and specificity is poorly understood. It is often assumed that as antibodies increase in affinity they also become more specific, and at a trivial level this is likely. A single high affinity antibody might only bind one or a few targets with strength. The serum response to T-dependent antigens is, however, a composite of antibodies from many clones, and it has long been known that ‘specific’ antibody responses produce antibodies that react with a variety of unrelated antigens [1]. As an antibody response progresses the number of V-regions or CDR3s used against some antigens may reduce [2], [3] although against others numbers may increase [4], and therefore while at a coarse level the repertoire may become more restricted, somatic hypermutation in germinal centers diversifies V-genes to such an extent that at the sequence level many cells have different receptors [2], [3], [5]–[7]. These different antibodies would each have slightly different antigen binding regions that may have specificity beyond that for the immunizing epitope [8], [9] and there is also evidence that somatic mutation can change antibody specificity altogether [10], [11]. The genetic diversification caused by new V-gene recruitment and somatic hypermutation has, therefore, the potential to expand the number of epitopes bound by a developing antibody response. Although generation of frank self-reactivity can lead to rapid apoptosis in germinal centres [12]–[14], and so defines an extreme limit of the diversification of specificity, the overall scope of reactivity produced during antibody maturation is currently little understood.

This issue is important, however, because it has a bearing on the related subjects of how broad the antibody reactivity induced by vaccine epitopes is, and similarly, the degree to which antibody responses to pathogen epitopes protect against variants of the epitope. With perfect specificity, an antibody response would not show binding to any epitope variants; with no specificity, a response would show binding to all possible non-self epitopes. Determining where on this scale of specificity *in vivo* responses lie, and how they change as the response progresses, is an aim of the study reported here.

## Results

To investigate how specificity changes during an antibody response serum IgG was screened at different times after immunisation with the HIV gp41 epitope ELDKWA [15], against a library of ELDKWA variants. To systematically introduce minimal changes in the epitope, and mimic how pathogen escape variants might manifest, a peptide library was created by synthesising all (except cysteine to avoid thiol reactions) single amino acid substitutions of the central LDKW sequence. ELDKWA was chosen as the antigen because it is short and so should not contain multiple independent epitopes, it is well characterised, derived from a real protein and the epitope is maintained in a linear peptide [15], [16]. Further, ELDKWA is a defined high affinity antibody epitope as it is the target of human monoclonal 2F5, which blocks HIV infection *in vivo* and *in vitro* [17]–[19]. We use 2F5 as a comparison antibody in this study.

### The anti-ELDKWA response shows affinity maturation

**Figure 1:**
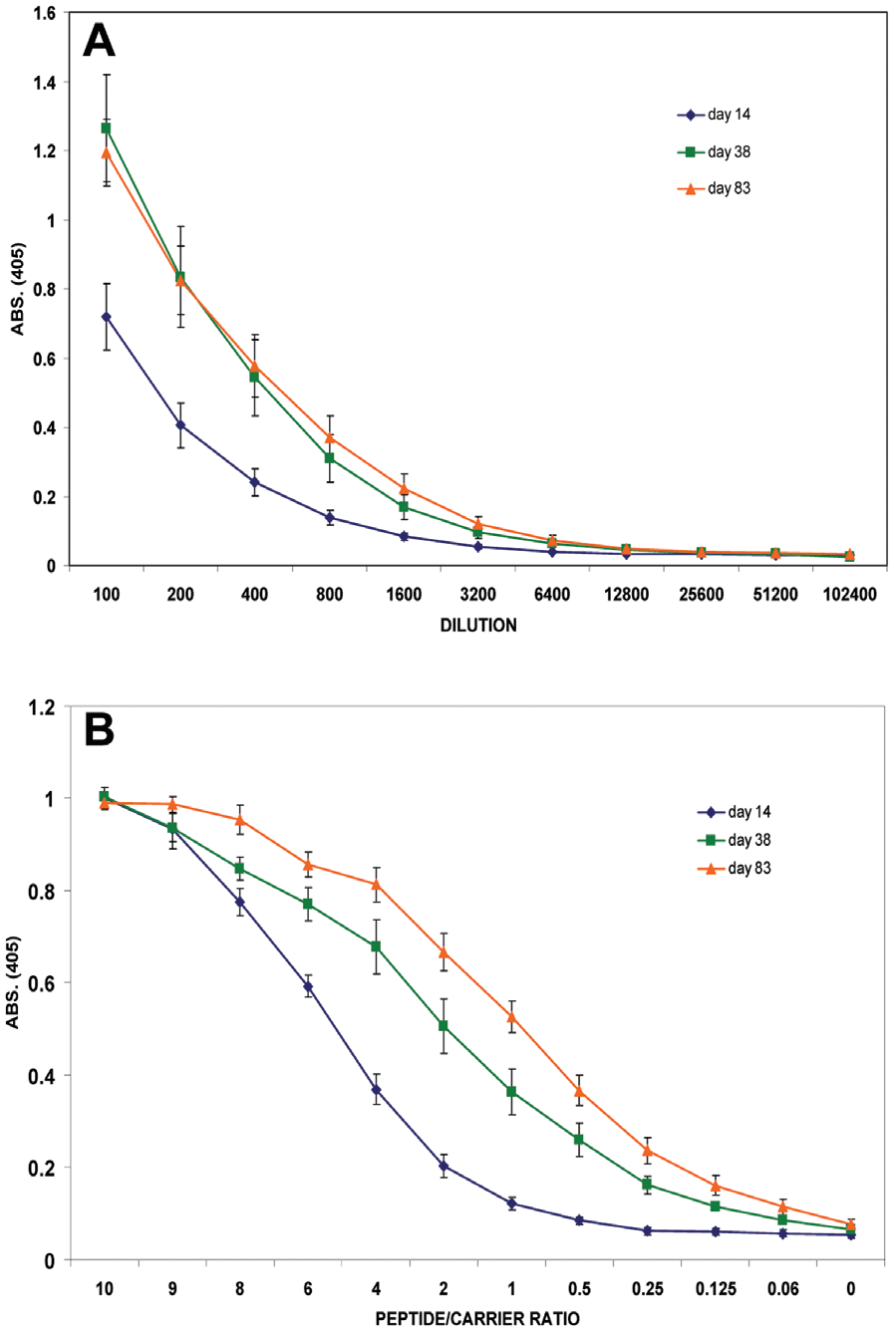
Anti-ELDKWA serum IgG titre and relative affinity. **A**, serum IgG titre 14 (blue), 38 (green), and 83 (brown) days after immunization. **B**, relative affinity of serum IgG with decreasing peptide/carrier conjugation ratio, after 14 (blue), 38 (green) and 83 (brown) days. Error bars: standard error of the mean, from 7 (day 14) or 8 (day 38, 83) total samples from two separate immunizations.

Eight mice were immunised with 50 ug of CELDKWAS peptide conjugated to KLH in alum, for each time-point. The n-terminal C was added for efficient crosslinking to carrier and also included with the c-terminal S residue, part of the native sequence, to avoid problems with antibodies specific for just the terminal residue (see methods). Serum was obtained 14, 38 and 83 days after CELDKWAS immunisation. These time-points were chosen to cover the period from the peak of the primary response to the time by which affinity maturation has occurred; for example see reference 21. Figure 1A shows the anti-peptide serum IgG titres for these time points. Titres rise from day 14 to day 38, and at day 83 show similar levels to day 38. To measure affinity maturation, sera were screened against a peptide/carrier titration, which discriminates for high affinity IgG binding [20], [21]. Figure 1B shows that the relative affinity of serum IgG for ELDKWA continues to rise over the time course of the experiment, consistent with previous observations that affinity maturation can continue for over 100 days after immunisation [21].

### Serum binding to ELDKWA variants

**Figure 2:**
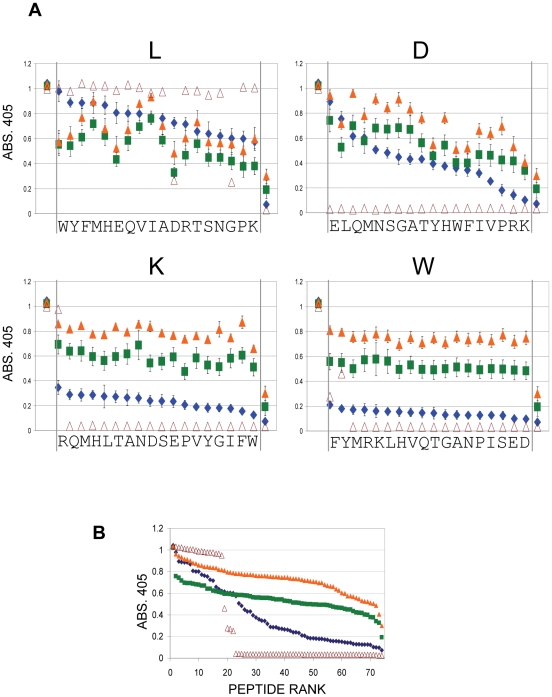
Reaction of anti-ELDKWA serum IgG with epitope variants. **A**, mean values of reactivity with epitope variants (coupling ratio 10∶1 ovalbumin), at a serum dilution that gives an absorbance of 1 against ELDKWA, after 14 (blue), 38 (green) and 83 (brown) days. Open brown triangles, 2F5 monoclonal. L, D, K, W denote the original amino-acid, small letters on x-axis denote the substitution in the variant. For clarity, values have been ranked according to the day 14 (blue) reading. First data point in each panel is the value for ELDKWA; last data point is for the randomized epitope sequence LAKDWE. Error bars: standard error of the mean, from 7 (day 14) or 8 (day 38, 83) total samples from two separate immunizations. **B**, ranked (separately for each time point) mean reactivity of anti-ELDKWA serum IgG against all 72 epitope variants and control after 14 (blue), 38 (green) and 83 (brown) days. Open brown triangles, 2F5 monoclonal.

All serum samples, from each time point after immunisation, were then used individually to screen the ELDKWA variants library containing the 72 possible (except cysteine) single amino-acid substitutions of LDKW. Serum from each individual mouse was used at the dilution that gave an IgG absorbance reading of 1 against the native peptide. This allows a comparison of the relative effects of substitutions on the binding of antibody from each time-point. The results are shown in Figure 2A. For the purpose of clarity the data for the substitutions at each particular position are shown separately. The data in each panel have been ranked according to the absorbance reading for each variant from day 14 serum. Readings for the native peptide and a CLAKDWE randomised control peptide are also included in each panel.

After 14 days serum IgG binding was heavily dependent on the K and W residues, as all substitutions in these positions severely diminished binding. Most D position substitutions also had a large effect on IgG binding whereas alterations at the L position had a lesser effect but, as for the D position, this depended on the particular substitution. After 38 days most D and all K and W substitutions were better tolerated; serum showed higher levels of binding to these variants than at day 14. Day 38 serum binding became more dependent on the L residue, however, as substitutions here reduced binding more than at day 14. Overall the binding of day 38 serum IgG to variants increased and became less dependent on any particular residue. This trend continued to day 83 when levels of IgG binding to all variants was greater than at day 38. The binding of total serum IgG to most single substitution variants of the epitope, therefore, increased markedly over time. The exception was for substitution at the L residue where the binding pattern was more complex. Binding of day 83 serum to about half the variants at this position recovered after the drop at day 38 to the levels seen at day 14.

Figure 2A also shows the binding of the anti-ELDKWA human monoclonal 2F5. 2F5 is known to have a core recognition sequence of D(K/R)W [16] and these data confirm this. This figure shows how intolerant the monoclonal antibody is of epitope variation, with only 3/54 DKW substitutions showing any binding; demonstrating a difference in binding between single antibodies and polyclonal sera. Figure 2B shows all the data points from Figure 2A ranked in value for each timepoint to show the overall trend of increasing binding to epitope variants.

### High affinity binding to epitope variants

It is possible that the increased binding to epitope variants is simply because antibody affinity for the native epitope has increased so much that single substitutions in it do not cause a loss of binding that can be detected here. With the high peptide to carrier coupling ratio used (10∶1) low as well as high affinity IgG would be detected. An epitope variation that reduced antibody-binding affinity by one or two logs from an initially high affinity may, therefore, still bind. To further investigate this issue and to test for high affinity binding to the variant sequences, the assay was repeated with peptide variants that have been coupled to carrier at low density, 1∶1 peptide: ovalbumin, chosen from the results in Figure 1B. At this coupling ratio only higher affinity IgG binding to peptide will be detected. Any binding to epitope variants will indicate a spreading of IgG specificity to these variants.

**Figure 3:**
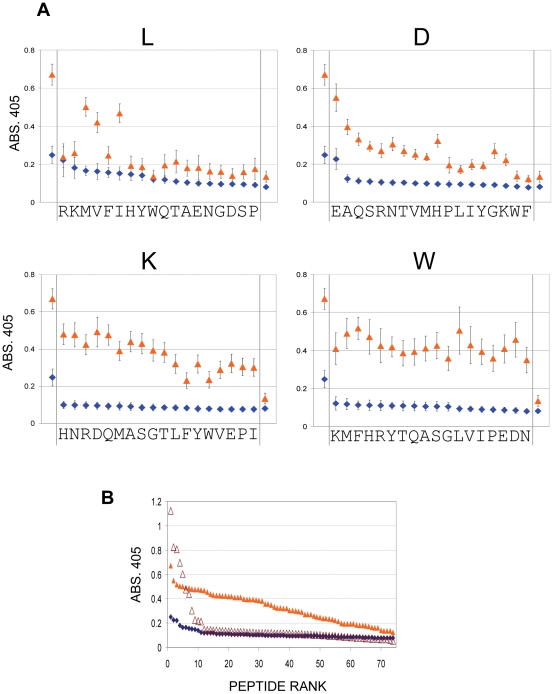
Higher affinity reaction of anti-ELDKWA serum IgG with epitope variants. **A**, mean values of reactivity with epitope variants (coupling ratio 1∶1 ovalbumin), at the serum dilution that gives an absorbance of 1 against ELDKWA coupled 10∶1 with ovalbumin, after 14 (blue) and 83 (brown) days. L, D, K, W denote the original amino-acid, small letters on x-axis denote the substitution in the variant. For clarity, values have been ranked according to the day 14 (blue) reading. First data point in each panel is value for ELDKWA; last data point is for the randomized epitope sequence LAKDWE. Error bars as for Figure 2A. **B**, ranked (separately for each time point) mean high affinity reactivity of anti-ELDKWA serum IgG against epitope variants (coupling ratio 1∶1 ovalbumin) after 14 (blue) and 83 (brown) days. Open brown triangles, 2F5 monoclonal.

For brevity, as affinity maturation is continuing until day 83 (Figure 1b) we have omitted data from the day38 time-point, to just show the total effect between the first and last time-points. Serum from day 14 and day 83 was screened against the variant peptide library, coupled at low density to ovalbumin, using the same dilutions as in Figure 2. These dilutions were used, because they were used in the peptide/carrier ratio titration, shown in Figure 1b, and give a good discrimination of high affinity binding when the peptide/carrier ratio is 1. The results are shown in Figure 3A. This figure shows that at day 83 serum IgG showed a significant level of higher affinity binding with most D, K, or W and some L substitutions. Day 14 sera showed little high affinity binding to any epitope variants, which is expected, as reactivity of serum with the native peptide at this coupling ratio is low (Figure 1B). Maturation of the response against ELDKWA is associated, therefore, with an increase in high affinity IgG binding to most epitope variants tested. Figure 3B shows all the data points ranked for each timepoint, and shows that there were greater levels of high affinity IgG binding to most variants at day 83 than there was binding to ELDKWA at day 14. Specificity, as defined by high affinity binding to variant sequences, has broadened after maturation of this immune response.

Figure 3B also shows the ranked values for high affinity binding of the monoclonal 2F5 to the epitope variants. The monoclonal showed high affinity binding at high levels to 6/72 variants with most of the rest showing no binding at all; this picture contrasts with the moderate levels of high affinity binding to most variants shown by the day 83 serum.

### High affinity binding related to the type of substitution in epitope variants

To further investigate the change in specificity of the maturing antibody response to ELDKWA, correlation between the level of high affinity binding to particular variants with the type of substitution in the variant was investigated. Some substitutions will have a greater effect on the epitope shape than others, and it would be informative to compare the degree of change in the epitope with the level of IgG binding. The most appropriate measure of the change induced by single amino-acid substitutions in protein sequences is the PAM250 score [22]. The PAM-250 matrix, which provides scores for every possible single amino-acid substitution, has been derived by comparing the amino acid sequences of a large set of closely related proteins to determine the frequencies of each particular amino-acid substitution between them. On the basis that substitutions have been evolutionarily selected, the frequency of a particular substitution reflects how functionally similar it is to the original amino-acid. Using the PAM-250 matrix to score differences in epitope sequences, therefore, provides a useful measure of the likelihood that change will be found in a natural epitope, as well as a measure of how much the substitution changes the structure/function of the epitope. Comparing these scores with the affinity of the antiserum against the substituted peptide will provide information on how affinity maturation changes the tolerance of antiserum binding to pathogen epitope changes that vary from small more likely changes to larger less likely ones.

The PAM-250 score for a substitution is usually presented as the log of the frequency that a particular change is found after 250 evolutionary steps. Positive scores are substitutions found more often than would be found randomly, and represent conservative changes that alter function less. Negative scores represent substitutions occurring less often than random and equate to non-conservative changes [28].

**Figure 4:**
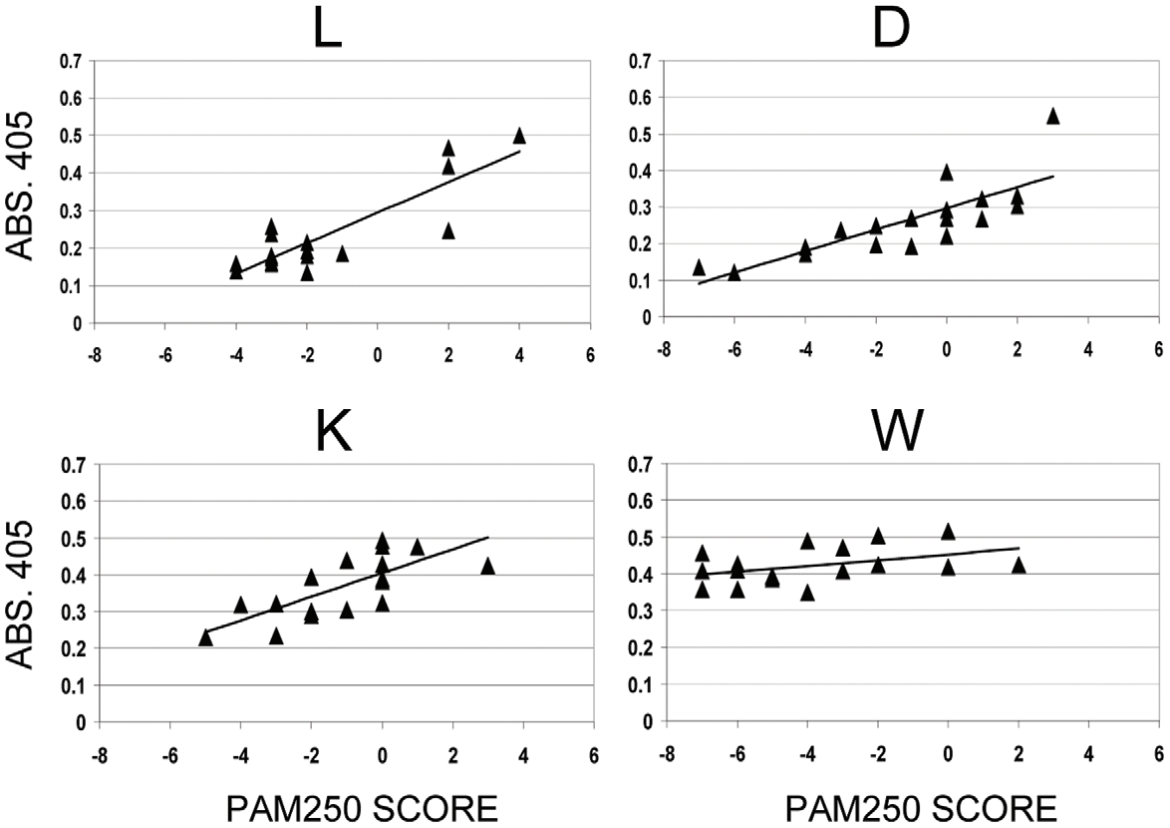
Correlation of serum IgG binding with PAM250 value of L, D, K and W substitutions. Mean values of high affinity serum IgG reactivity with epitope variants (from Figure 3A) plotted against the PAM250 value of the substitution in each variant. L, D, K and W refer to the particular epitope residue substituted.

The IgG binding values from Figure 3A were plotted against the PAM250 score for each particular substitution (Figure 4). This figure shows a positive correlation overall between IgG binding and PAM250 score, in particular for substitutions at L, D and K, implying that serum IgG reactivity has a bias towards conservative changes in epitope. This bias, the slope of the trend-line; and also whether the trend line intercepts the baseline reading of 0.13 (binding of day 83 serum to the LAKDWE control peptide) defines the specificity of the serum, by showing the degree of change that can be tolerated at any particular residue in the epitope.

The most non-conservative substitutions in the L and D residues show baseline absorbance readings in Figure 4, and so loss of high affinity IgG binding. This observation, combined with the positive correlation of absorbance and PAM250 score, indicate that anti-ELDKWA serum was more sensitive to the type of substitution at these residues than at K and W. Conversely, at the K and W residues, despite the overall trend toward higher binding to epitopes with conservative changes, there was significant high affinity IgG binding to variants with highly non-conservative substitutions. In effect, the serum would tolerate more than a single substitution at these positions whilst still showing high affinity IgG binding.

High affinity IgG binding did not show much bias for particular substitutions at the W residue as the absorbance/PAM250 correlation trend-line is almost flat. Most substitutions of tryptophan have negative PAM250 scores; with the exception of W to R it cannot be conservatively substituted. This could explain why substitutions of this residue showed very little correlation of binding with the PAM250 score. As almost all substitutions at this residue result in a gross change, serum IgG may not discriminate between particular substitutions even though they contain different residues.

## Discussion

By screening serum from a maturing antibody response against a library of peptides we have shown that an increase in titre and affinity of IgG is accompanied by an increase in high affinity binding to epitope variants; that at the scale of single amino acid differences, antibody maturation causes a broadening of antiserum specificity, to include non-conservatively substituted epitope variants. There were greater levels of high affinity IgG binding to 48 of 72 variants at day 83 than there were to the native epitope at day 14. This spread of specificity is in marked contrast to that seen for a monoclonal antibody.

By correlating antibody binding with the degree of change (PAM250 score) in the epitope, and considering the intersection of the trend-line in Figure 4 with baseline readings, we can define the specificity of the antiserum. After maturation of the response against ELDKWA, high affinity serum specificity has spread to certain substitutions in the L and D residue, but beyond single substitutions in the K and W residues, as any changes here do not completely abolish antibody binding and so additional changes may be tolerated.

Single substitutions do not tend to cause the catastrophic loss of antiserum binding as they mostly do with the monoclonal antibody, but rather, a graduated reduction inpart dependant on the degree of similarity of the substitution to the native residue. This, as discussed below, is likely to be an effect of the polyclonality of the antiserum, and if this provides enhanced protection against variant pathogens it is a good reason why humoral responses do not rapidly become monoclonal.

Although we have only analysed single substitutions in a short epitope – and this therefore defines the scale within which we observe a spread of specificity – the level of higher affinity binding observed against non-conservative substitutions is still surprising. Although the changes described here might be described as diversification of ‘fine specificity’ [4] this cannot be simply assumed. Changes that affect only fine specificity would not alter the binding of an antibody to any other epitope in the set of all possible epitopes, and this is difficult to determine. Given that antibody maturation can involve recruitment of new VDJ combinations as well as somatic mutation, it is conceivable that an ongoing antibody response could introduce entirely new binding specificities.

The observation that low affinity clones can initiate germinal centres [23] and then undergo somatic mutation regardless of their initial affinity [24] suggests that clones with many different V-genes participate in the early primary response. After this point, the selective forces acting on clones that mutate their antibody genes are the need to continue binding antigen [25] and the deletion of self-reactive clones [12]–[14]. The pressure to continue binding antigen, at increasing affinity, might place tight constraints on the permissible variability in the antigen-binding region, but these constraints will vary depending on the V-gene used and epitope type and they are currently unknown. Within these constraints, antigen-binding regions would be free to evolve and experience neither positive nor negative selective pressure on mutations at residues not crucially involved in binding. This process would cause a diversification of specificity related to the number of genetically distinct clones. The data presented here are consistent with this hypothesis as, at the scale of single amino acid substitutions in epitopes, a broadening of specificity evolves during antibody maturation. The contrast between antiserum and monoclonal binding profiles support the notion that this effect is caused by increasing clonality rather than increasing affinity. Further, the notion that increased binding to epitope variants is caused simply by an increase in affinity is inconsistent with the data, as less IgG bound to variants substituted at the L residue at days 38 and 83 than at day 14, even though the IgG affinity for ELDKWA had increased at these time points.

The results presented here are consistent with the postulate that antiserum maturation generates new specificities that can bind pathogen escape variants. Many potential pathogen epitope variants will manifest as single amino-acid substitutions, and we suggest that many of these are likely to be covered by the maturing antiserum so would never become established. One might expect there to be a strong evolutionary pressure on antibody diversification in the primary response to allow cover for pathogen escape variants as well as increasing the affinity of antibodies against the priming epitope, although the possible selective forces driving this process are not understood. There is also the further risk of generating self-reactivity and this makes this area complex and difficult to study *in vivo*. It is particularly interesting, therefore, that a recent study has reported that some memory clones stimulated by infection with West Nile Virus in mice, have a higher affinity for variants of the epitope present on the immunising virus [29]. As it is known that high affinity clones arise earlier in primary responses than germinal centres regress [30], this observation supports the idea that ongoing somatic mutation aids in the diversification of antibody specificity to allow variant epitope binding in the manner reported here.

## Methods

### Ethics Statement

All experimental procedures were approved by the U.K. Home Office under license 70/05954 and had UCL ethics committee approval, Ref: 0897 and were performed in the Institute of Child Health licensed animal facility.

### Antigen and Immunization

CELDKWAS peptide was obtained from Sigma-Aldrich, U.K. and coupled at saturation to maleimide activated KLH (Pierce/Thermo Scientific, U.K.) according to manufacturers instructions. Female, 8 week-old BALB/c mice (Harlan, U.K.) were immunized intra-peritoneally with 50 ug alum precipitated antigen. Serum was collected 14, 38 and 83 days after immunization. Mice were immunized in groups of four for each time point, twice.

### Peptide Library

The peptide array consisting of the 72 possible (excepting C) single amino-acid substitution variants of the central LDKW sequence of CELDKWA was obtained from Mimotopes, U.K. and also included the native sequence and the randomized control sequence CLAKDWE.

The c-terminal S residue was deliberately included in the immunizing antigen and omitted in the screening peptides because sera against short peptides can contain antibodies against just the terminal residue [26]; these would mask the effects of LDKW sequence variation on antibody binding in this assay. Each peptide was coupled at saturation to maleimide-activated ovalbumin (Pierce/Thermo Scientific, U.K.) according to manufacturers instructions and purified by dialysis.

### Peptide/Carrier Ratio

To alter the peptide/carrier ratio from 10 moles peptide per mole of ovalbumin when coupled at saturating peptide concentration, CELDKWA type peptides were diluted the appropriate molar amount with the HIV TAT peptide GGGGYGRKKRRQRRRGC and then coupled at saturating peptide concentration. The same batch of maleimide-activated ovalbumin was used in all experiments involving lowered peptide/carrier ratios.

### ELISA

All ELISAs were performed according ref. 27, using alkaline phosphatase conjugates of anti-mouse or anti-human IgG (Sigma-Aldrich, U.K.) with pNPP substrate (Sigma-Aldrich, U.K.). Wells were coated overnight at room temperature with 2 ug ml−1 peptide/ovalbumin conjugates, incubated for 4 hours at room temperature with serum dilutions, 2 hours with alkaline phosphatase conjugates and 1 hour with pNPP substrate. All plates for a particular screening series (10∶1 coupling ratio or 1∶1 coupling ratio) were made at the same time and stored according to ref. [27].

## References

[1] Sperling R, Francus T, Siskind GW (1983). Degeneracy of Antibody Specificity.. J Immunol.

[2] Kuppers R, Zhao M, Hansmann ML, Rajewsky K (1993). Tracing B cell development in human germinal centres by molecular analysis of single cells picked from histological sections.. EMBO J.

[3] Jacob J, Przylepa J, Miller C, Kelsoe G (1993). In situ studies of the primary immune response to (4-hydroxy-3-nitrophenyl)acetyl. III. The kinetics of V region mutation and selection in germinal center B cells.. J Exp Med.

[4] Kalinke U, Bucher EM, Ernst B, Oxenius A, Roost HP (1996). The role of somatic mutation in the generation of the protective humoral immunse response against vesicular stomatitis virus.. Immunity.

[5] Jacob J, Kelsoe G, Rajewsky K, Weiss U (1991). Intraclonal generation of antibody mutants in germinal centres.. Nature.

[6] Berek C, Berger A, Apel M (1991). Maturation of the immune response in germinal centers.. Cell.

[7] Weiss U, Zoebelein R, Rajewsky K (1991). Accumulation of somatic mutants in the B cell compartment after primary immunization with a T cell-dependent antigen.. Eur J Immunol.

[8] Schildbach JF, Panka DJ, Parks DR, Jager GC, Novotny J (1991). Altered hapten recognition by two anti-digoxin hybridoma variants due to variable region point mutations.. J Biol Chem.

[9] Fish S, Fleming M, Sharon J, Manser T (1991). Different epitope structures select distinct mutant forms of an antibody variable region for expression during the immune response.. J Exp Med.

[10] Rudikoff S, Giusti AM, Cook WD, Scharff MD (1982). Single amino acid substitution altering antigen-binding specificity.. Proc Nat Acad Sci.

[11] Diamond B, Scharff MD (1984). Somatic mutation of the T15 heavy chain gives rise to an antibody with autoantibody specificity.. Proc Nat Acad Sci.

[12] Rathmell JC, Cooke MP, Ho WY, Grein J, Townsend SE (1995). CD95 (Fas)-dependent elimination of self-reactive B cells upon interaction with CD4+ T cells.. Nature.

[13] Pulendran B, Kannourakis G, Nouri S, Smith KG, Nossal GJ (1995). Soluble antigen can cause enhanced apoptosis of germinal-centre B cells.. Nature.

[14] Han SH, Zheng B, Porto JD, Kelsoe GJ (1995). In situ studies of the primary immune response to (4-hydroxy-3-nitrophenyl)acetyl. IV. Affinity-dependent, antigen-driven B cell apoptosis in germinal centers as a mechanism for maintaining self-tolerance.. J Exp Med.

[15] Muster T, Steindl F, Purtscher M, Trkola A, Klima A (1993). A conserved neutralizing epitope on gp41 of human immunodeficiency virus type 1.. J Virol.

[16] Tian Y, Ramesh CV, Ma X, Naqvi S, Patel T (2002). Structure-affinity relationships in the gp41 ELDKWA epitope for the HIV-1 neutralizing monoclonal antibody 2F5: effects of side-chain and backbone modifications and conformational constraints.. J Peptide Res.

[17] Conley AJ, Kessler JA, Boots LJ, Tung JS, Arnold BA (1994). Neutralization of divergent human immunodeficiency virus type 1 variants and primary isolates by IAM-41-2F5, an anti-gp41 human monoclonal antibody.. Proc Natl Acad Sci.

[18] Muster T, Guinea R, Trkola A, Purtscher M, Klima A (1994). Cross-neutralizing activity against divergent human immunodeficiency virus type 1 isolates induced by the gp41 sequence ELDKWAS.. J Virol.

[19] Mascola JR, Lewis MG, Stiegler G, Harris D, VanCott TC (1999). Protection of Macaques against pathogenic simian/human immunodeficiency virus 89.6PD by passive transfer of neutralizing antibodies.. J Virol.

[20] Herzenberg LA, Black SJ, Tokuhisa T, Herzenberg LA (1980). Memory B cells at successive stages of differentiation. Affinity maturation and the role of IgD receptors.. J Exp Med.

[21] Takahashi Y, Dutta PR, Cerasoli DM, Kelsoe G (1998). In situ studies of the primary immune response to (4-hydroxy-3-nitrophenyl)acetyl. V. Affinity maturation develops in two stages of clonal selection.. J Exp Med.

[22] Pearson WA, Doolittle R (1990). Methods in Enzymology,.

[23] Dal Porto JM, Haberman AM, Kelsoe G, Shlomchik MJ (2002). Very low affinity B cells form germinal centers, become memory B cells, and participate in secondary immune responses when higher affinity competition is reduced.. J Exp Med.

[24] Yang Shih T-A, Meffre E, Roederer M, Nussenzweig MC (2002). Role of BCR affinity in T cell-dependent antibody responses in vivo.. Nature Immunol.

[25] MacLennan ICM (1994). Germinal Centres.. A Rev Immunol.

[26] Palfreyman JW, Aitcheson TC, Taylor PJ (1984). Guidelines for the production of polypeptide specific antisera using small synthetic oligopeptides as immunogens.. Immunol Meth.

[27] Hornbeck P, Coligan JE, Kruisbeek AM, Margulies DH, Shevach EM, Strober W (1991). Current Protocols in Immunology, chapter 2, eds..

[28] Dayhoff MO, Schwarz RM, Orcutt BC (1978). A model of evolutionary change in proteins,. An Atlas of Protein Sequence and Structure, National Biomedical Research Foundation.

[29] Purtha WE, Tedder TF, Johnson S, Bhattacharyal D, Diamond MS (2011). Memory B cells, but not long-lived plasma cells, possess antigen specificities for viral escape mutants.. J Exp Med.

[30] Smith KG, Light A, Nossal GJ, Tarlinton DM (1997). The extent of affinity maturation differs between the memory and antibody-forming cell compartments in the primary immune response.. EMBO J.

